# A Dynamic Study of Protein Secretion and Aggregation in the Secretory Pathway

**DOI:** 10.1371/journal.pone.0108496

**Published:** 2014-10-03

**Authors:** Maria Francesca Mossuto, Sara Sannino, Davide Mazza, Claudio Fagioli, Milena Vitale, Edgar Djaha Yoboue, Roberto Sitia, Tiziana Anelli

**Affiliations:** 1 Division of Genetics and Cell Biology, IRCCS Ospedale San Raffaele, Milan, IT; 2 Department of Biosciences, Università degli Studi di Milano, Milan, IT; 3 Università Vita-Salute San Raffaele, Milan, IT; 4 Experimental Imaging Center, IRCCS Ospedale San Raffaele, Milan, IT; University of Pittsburgh, United States of America

## Abstract

Precise coordination of protein biogenesis, traffic and homeostasis within the early secretory compartment (ESC) is key for cell physiology. As a consequence, disturbances in these processes underlie many genetic and chronic diseases. Dynamic imaging methods are needed to follow the fate of cargo proteins and their interactions with resident enzymes and folding assistants. Here we applied the Halotag labelling system to study the behavior of proteins with different fates and roles in ESC: a chaperone, an ERAD substrate and an aggregation-prone molecule. Exploiting the Halo property of binding covalently ligands labelled with different fluorochromes, we developed and performed non-radioactive pulse and chase assays to follow sequential waves of proteins in ESC, discriminating between young and old molecules at the single cell level. In this way, we could monitor secretion and degradation of ER proteins in living cells. We can also follow the biogenesis, growth, accumulation and movements of protein aggregates in the ESC. Our data show that protein deposits within ESC grow by sequential apposition of molecules up to a given size, after which novel seeds are detected. The possibility of using ligands with distinct optical and physical properties offers a novel possibility to dynamically follow the fate of proteins in the ESC.

## Introduction

To achieve their native structure, secretory and membrane proteins exploit the vast array of chaperones and enzymes that reside in the endoplasmic reticulum (ER), the port of entry into the secretory compartment. Here, they undergo stringent quality control [1], [2]: only properly folded and assembled proteins are given the green light and proceed along the secretory pathway. Proteins that fail to attain their native state are eventually retro-translocated to the cytosol for proteasomal degradation. Not all proteins entering the ER are secreted or directed to the plasma membrane. Even if in some conditions the flux of cargo can become intense, resident proteins stop at the desired stations to maintain organelle identity and guarantee function. For instance, soluble ER residents are retrieved from downstream stations via KDEL-Receptors [3].

The sophisticated systems deployed by cells to regulate this intense traffic and prevent dangerous jams in ESC are unfortunately not fully reliable. Sometimes, an overzealous quality control can cause systemic loss of function diseases preventing the transport of mutants that are nonetheless active. Unless promptly degraded, moreover, these can condense in ESC and cause gain of function diseases [4].

Secretory IgM are complex polymers [5] whose biogenesis occurs stepwise in ESC [6]. Like other unassembled Ig-H chains, secretory µ (µ_s_) interact with BiP via their first constant domain (C_H_1). Assembly with Ig-L displaces BiP, and µ2L2 complexes are then slowly polymerized [7]. When C_H_1 is lacking, µΔC_H_1 accumulate in a detergent insoluble form within dilated ESC cisternae, also called Russell Bodies (RB) [8], [9] providing a suitable model system for Heavy Chain Disease (HCD [10] and references therein) and ER storage disorders (ERSD [11]). We recently identified some of the factors that modulate µΔC_H_1 condensation in living cells. For instance, over-expression of ERp44, a multifunctional chaperone that mediates thiol-dependent quality control of IgM subunits and other clients [12], [13], stimulated the accumulation of µΔC_H_1 in RB [14].

To learn more about how cells handle different proteins in ESC, we generated different chimeric proteins containing a Halotag (Halo) derived from a *Rhodococcus rhodochrous* Haloalkane dehalogenase whose active site has been engineered to covalently bind fluorescently-labelled chloro-alkane derivatives [15], [16]. With respect to more conventional live-cell labelling based on fluorescent proteins the Halotag post-translational labelling system has several advantages. First, it allows to using organic dyes such as TMR (tetramethyl-rhodamine) or R110, that are brighter and more photostable than fluorescent proteins [17] and whose fluorescence is relatively pH-insensitive [18]. By selecting suitable ligands the same tag can be used for live cell microscopy, immunofluorescence, Western Blotting, protein purification and co-precipitation assays [16], [17], [19]–[26]. Moreover, the Halotag allows following the accumulation and/or the degradation of the protein of interest by two-color pulse/chase experiments with high temporal resolution [27]. Lastly, the Halotag has the advantage of not possessing glycosylation sites, that could affect folding and transport of the chimeric proteins in the secretory compartment.

Another hybrid system, based on small molecules able to covalently bind genetically specified proteins, is the tetracysteine biarsenical system [28]. Unfortunately, due to the oxidative environment of the ER, this system cannot be applied to the study of secretory proteins.

In this work, after confirming that Halo folds and maintains its activity in ESC without grossly perturbing the fate of the target protein, we followed the condensation of Halo-µΔC_H_1 in ESC and analysed the growth and mobility of the resulting RB *in vivo* exploiting the property of covalently binding ligands coupled to different fluorochromes. Moreover, by appending the Halotag to short- and long-lived ER residents and to transport competent molecules, we show that it is possible to follow protein degradation and secretion bypassing classic radioactive pulse and chase techniques. Thus, Halo is a versatile non-invasive tool to follow key events in the secretory pathway.

## Results and Discussion

### Following protein aggregation in the ER at single cell level

**Figure 1:**
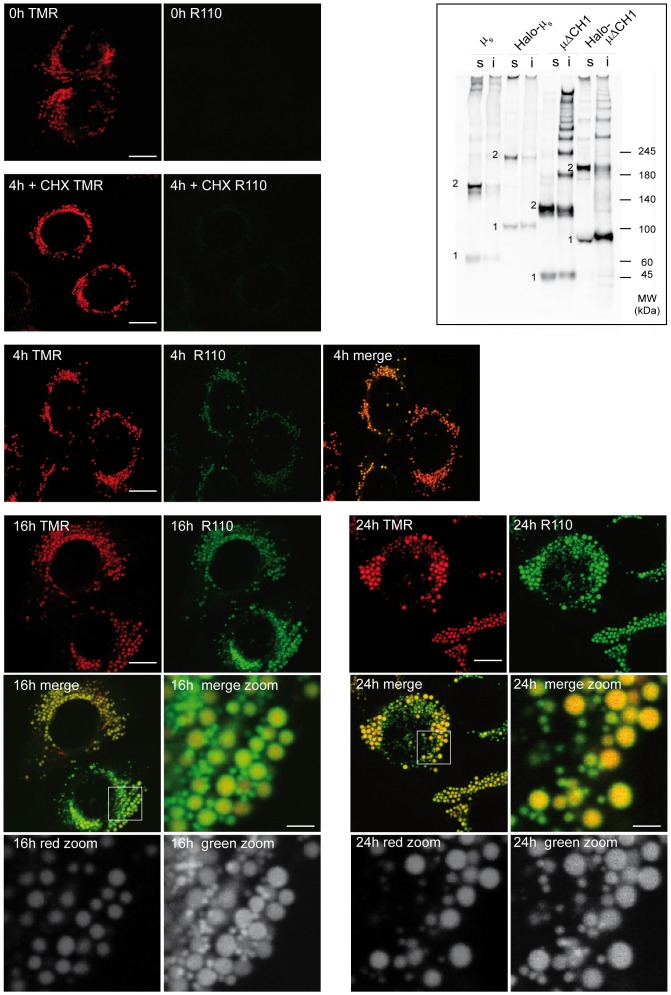
Halotag can be used to follow the aggregation/condensation of a protein in the ER. HeLa cells expressing Halo-µΔCH1 were labelled for 24 hours with the red TMR ligand (5 µM) in complete medium. After extensive washings, cells were incubated with the green ligand R110 (5 µM) for the indicated times. To control saturation, cells were treated with cycloheximide (0.5 mM) for the last 2 hours of the first TMR incubation, and then during the incubation with R110 for 4 hours. Cells were then fixed in paraformaldheyde and analyzed by confocal microscopy. Images are shown as confocal slices. With the progression of the chase, the green signal co-localizes with the pre-existing red signal and newly formed aggregates (only green) can be visible. Pre-existing red aggregates grow by apposition of newly synthetized proteins which can be labeled in green. (Bar: 5 µm). Single channel signals in gray scale are shown for the thumbnails (Bar: 1 µm). **Inset**: HeLa cells transiently expressing Halo-µΔC_H_1 or Halo-µ_s_ were lysed, and aliquots from soluble (s) and insoluble (i) fractions resolved under non-reducing conditions on a 3–8% polyacrylamide gradient gel. Nitrocellulose membranes were decorated with anti-µ. Bands indicated with 1 represent monomeric proteins, while bands indicated with 2 represent homodimers.

**Figure 2:**
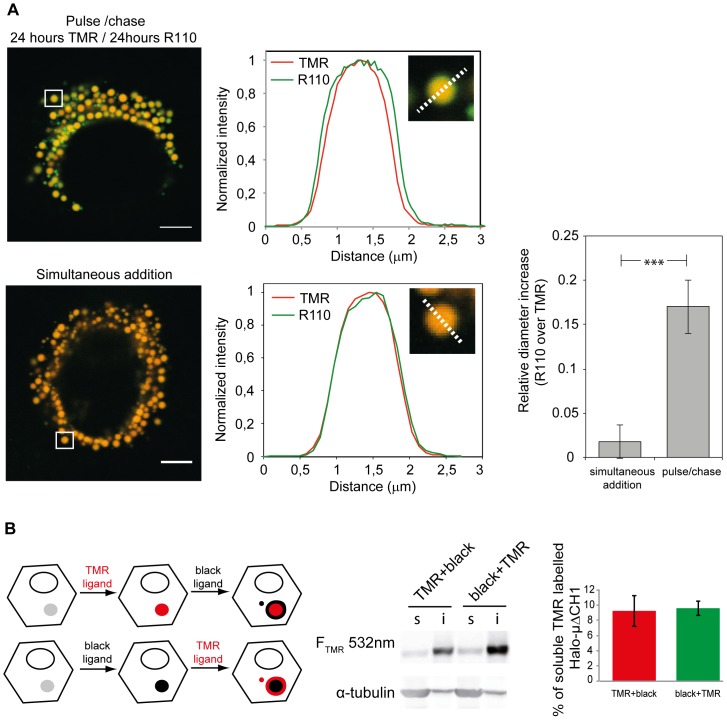
Visualising the growth of RB. **A** *Quantification of cluster growth by pulse-chase*. Images were acquired as described above and analyzed with custom-written Matlab routines to quantify the size of the clusters in the two channels. When the two ligands are added in sequence (with a 24 hours chase) an average 16%+/−3% (SEM) increase in the cluster size is observed, while no difference in size can be measured when the two ligands are added simultaneously. For the pulse and chase experiment 9 cells and 99 clusters were examined. For the simultaneous addition of the two ligands 9 cells and 101 clusters were analyzed. Bar: 5 µm. **B** *Distribution of old and young Halo-µΔC_H_1 molecules*. HeLa cells transiently expressing Halo-µΔC_H_1 were subjected to fluorescent pulse-chase assays: 24 hours with the TMR ligand and 24 h with the non-labelled ligand (TMR+black) or 24 hours with non-labelled ligand and 24 hours with the TMR ligand (black+TMR). Halo-ligands were used at 5 µM. Aliquots of soluble (s) and insoluble (i) material were then resolved under reducing conditions on a 10% polyacrylamide gel. After transferring to nitrocellulose, the signal of the TMR ligand was collected using fluorescence technology by FLA900 Starion. Densitometric quantifications are shown on the right. Average of 3 independent experiments +/− standard deviation.

When deleted of the C_H_1, Ig-µ chains display a strong tendency to become detergent insoluble and form RB [9]. To follow the biogenesis of RB *in vivo*, we inserted the Halo tag between the variable and the second constant domain of µΔC_H_1. Halo-µΔC_H_1 oligomerized and displayed detergent solubility similar to its untagged counterpart (Figure 1, inset). This behaviour suggests that even in the oxidising ER environment the Halotag, which contains two free cysteines, does not form aberrant intra- or inter-chain disulphide bonds, as confirmed also by the electrophoretic mobility of an ER-located Halotag alone under reducing and non-reducing conditions (Figure S1). We thus investigated how Halo-µΔC_H_1 aggregates grow in living cells by designing fluorescent pulse-chase experiments. The rationale of these assays is to use sequentially differently coloured ligands (TMR and R110) to differentially label pre-existing ‘old’ molecules and newly made ‘young’ ones. HeLa cells expressing Halo-µΔC_H_1 were labelled 24 hours with the red ligand (TMR) and washed before addition of the green ligand (R110). After different time intervals, cells were fixed and analysed by confocal microscopy. As the incubation time with the R110 ligand increases, green staining becomes progressively more intense (Figure 1). When protein synthesis was blocked by the addition of cycloheximide 2 hours before and 4 hours during the treatment with the second ligand, no green staining was detectable. This confirmed that all the pre-existing molecules were labelled with the red ligand and that no ligand exchange between molecules occurs in living cells. Hence, our labelling protocol can be used to discriminate between pre-existing and newly synthesized molecules. After 4 hours, green staining almost completely co-localized with the pre-existing RB. As synthesis continued, bigger yellowish dots became evident, consistent with the accumulation of newly made molecules next to pre-existing aggregates, which progressively grew in size. At later times, a green circle could be observed around most pre-existing red aggregates: these onion-like structures imply that roundish deposits of pre-existing molecules grow by sequential addition of layers containing newly made proteins. Together with the old growing aggregates, new green small dots can be observed at later times, probably representing new seeds of protein condensation. Very few red-only aggregates can be seen, indicating that most pre-existing structures act as seeds. We measured the increase in size of the aggregates after 24 hours of growth in the presence of the green ligand: we observed a relative diameter increase of about 16% (Figure 2A, upper panel). As a control, no difference in the diameter of the aggregates was observed when cells were incubated simultaneously with both red and green ligands (Figure 2A, lower panel). The possibility of detecting RB by Immunofluorescence correlates with the accumulation of µΔC_H_1 chains in the detergent insoluble fractions. These derive from the condensation of proteins that fail to be degraded by ERAD [9], [14]. Interestingly, young molecules distribute into soluble and insoluble fraction as the old ones, indicating that independently on the aggregation-step the Halo-µΔC_H_1 molecule reaches equilibrium between soluble and insoluble species (Fig. 2B). Hence also the smaller green dots observed in fluorescence contain insoluble molecules.

**Figure 3:**
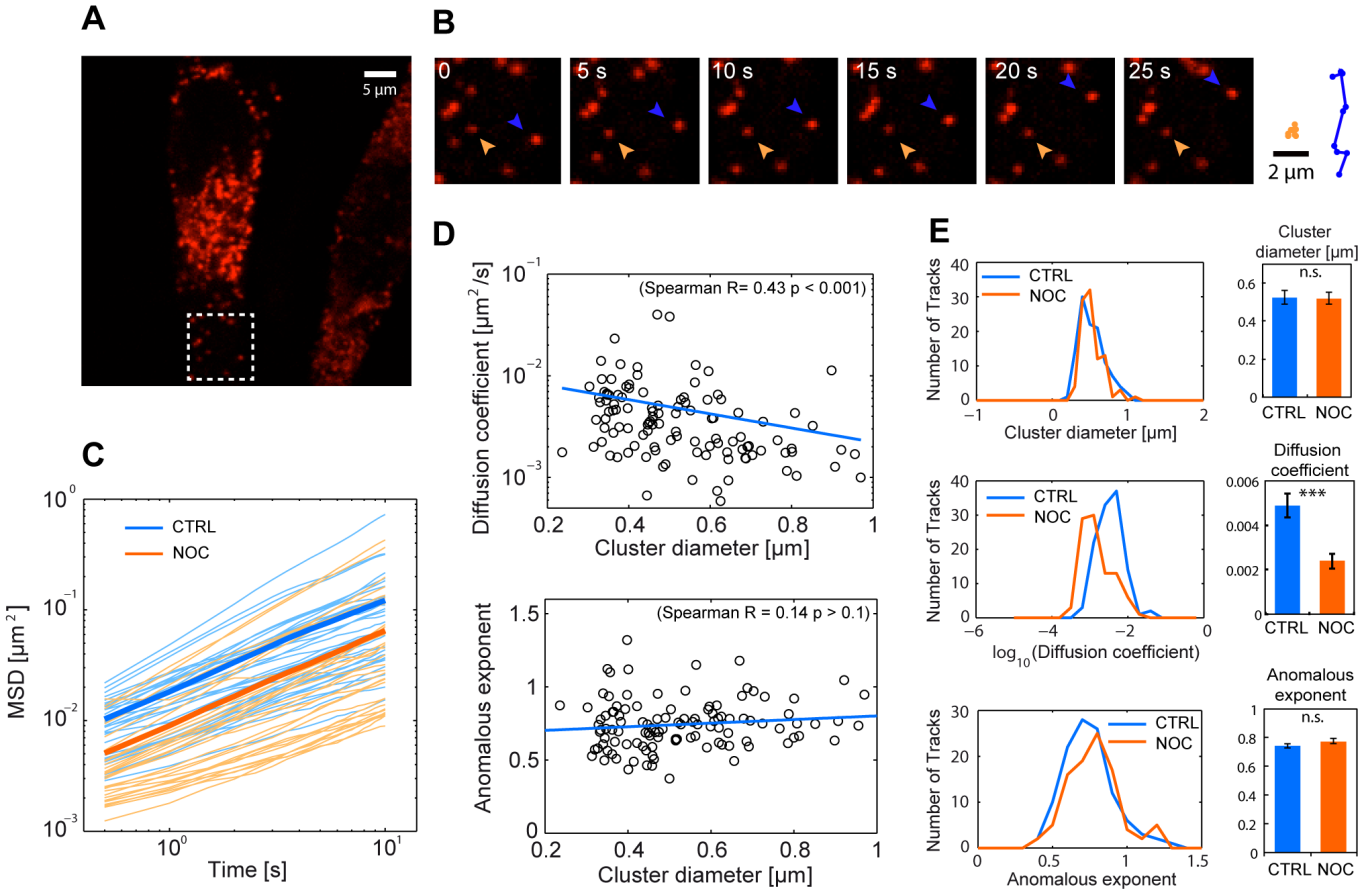
Live-imaging of ER protein aggregation. **A** HeLa cells expressing Halo-µΔC_H_1 were labeled for 24 h with 5 µM TMR. Living cells were then imaged by confocal microscopy at a frame rate of 2 images/s. **B** Aggregates display a heterogeneous dynamic behavior with some clusters moving directionally (orange arrowhead) and others showing confined diffusion (blue arrowhead). **C** We tracked individual clusters in 8 cells and we selected tracks that were longer than 150 frames (115 tracks) and we plotted individual MSD curves (orange thin lines) and their average (thick line). Treatment of cells with 0.3 µg/ml Nocodazole for 15 hours (blue lines) decreased of the mobility of the aggregates. **D** The diffusion coefficient D and the anomalous exponent α were calculated from the MSD curves obtained from the untreated samples and plotted against the estimated diameter of the clusters. On average we measured α = 0.74±0.02 and average diffusion coefficient *D* = 0.0045±0.0005 µm^2^/s. A value of α<1 is representative of anomalous subdiffusion, probably due to obstacles constraining the mobility of the clusters. While the anomalous exponent did not seem to depend on the cluster size, the diffusion coefficient was found to inversely correlate with the particle diameter. **E** We compared the distribution of measured particle sizes, diffusion coefficients and anomalous exponents for the untreated and the Nocodazole treated samples: the diffusion coefficient was the only parameter significantly affected by the disruption of the cytoskeleton (error bars: Mean ± SEM).

Next, we analysed the movement of Halo-µΔC_H_1 aggregates by tracking the individual RB in living cells by confocal live-cell microscopy (Figure 3A–B). For each of the 115 tracks extracted from 8 movies we measured the mean-square displacements (MSD) as function of time (Figure 3C) and we fitted the first 20 points of the MSD curves with a model for anomalous diffusion, *MSD* = 4*Dt ^α^* to obtain estimates for the short-term diffusion coefficient *D* and for the anomalous diffusion exponent α. In general, the tracks displayed anomalous subdiffusion, representative of constrained motion, with an average anomalous exponent α = 0.74±0.02 and average diffusion coefficient *D* = 0.0045±0.0005 µm^2^/s. This value is significantly lower than the diffusion coefficients of soluble ER proteins [29]–[32], further confirming that Halo-µΔC_H_1 clusters display constrained mobility. Interestingly, RB diffuse with a similar anomalous exponent independently of their size, while as expected larger (and potentially older) clusters displayed a reduced diffusion coefficient (Figure 3D). We also observed a few small clusters moving directionally (blue arrowed in Figure 3B), suggesting that the microtubule network might play a role in the dynamic behaviour of the clusters. We therefore quantified the mobility of the clusters following the depolymerization of microtubules. Upon treatment with Nocodazole the Halo-µΔC_H_1 clusters displayed comparable size to the untreated samples, but their diffusion coefficients were 0.5x smaller on average (Figure 3E), confirming that the motion of the clusters is at least partially associated to the microtubule network.

### Following the degradation of an ER resident protein

**Figure 4:**
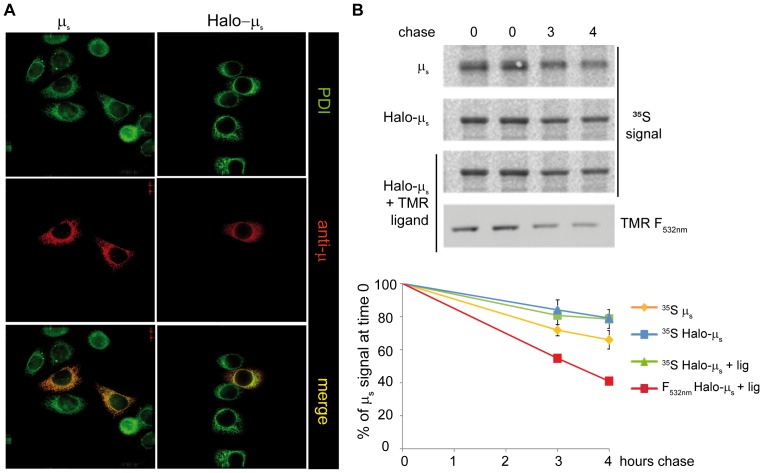
Exploting Halotag to study ERAD. **A** *Halo-tagging does not alter the localization of unassembled Ig-µ_s_*. HeLa cells were transiently transfected with µ_s_ or Halo-µ_s_ as indicated, fixed with PFA and stained with antibodies specific for PDI or Ig-µ. In this experiment, transfection efficiency was about 25%. Bar: 5 µm. **B** *The Halotag with or without bound ligand does not interfere with µ_s_ degradation*. 48 hours after transfection, HeLa cells expressing µ_s_ or Halo-µ_s_ cells were pulsed for 10 minutes with ^35^S aminoacids and chased in the presence of 5 mM DTT for the indicated time points, to accelerate degradation [35]. One sample (Halo-µ_s_+ligand) was pre-treated with TMR (2.5 µM) before and during the radioactive pulse. Aliquots from the lysates corresponding to 10^6^ cells for each time point were precipitated with anti-µ, resolved under reducing conditions and transferred to nitrocellulose membranes which were first developed for the ^35^S signal and then for TMR fluorescence (532 nm). Densitometric quantification was performed by ImageJ. The graph shown in the bottom panel represents the percentage of the total µ present at time 0 remaining at individual times.

In the absence of Ig-L, orphan µ_s_ are retained in the ER via interactions with BiP [7]. After a lag that varies amongst different substrates and/or cell types, they are retro-translocated into the cytosol and eventually degraded by proteasomes [33]–[35]. Like its untagged counterpart, Halo-µ_s_ is also retained in the ER, as indicated by its co-localization with the ER chaperone PDI (Figure 4A). Also in this case, the Halotag does not interfere with oligomer formation or solubility of the tagged protein (Figure 1, inset).

The standard approach to study protein degradation is based on radioactive pulse and chase experiments [36], limited by the restrictions of using radioactive isotopes and the difficulties in analysing the phenomenon at the single cell level. The use of cycloheximide chases [37] is restricted to short-lived proteins, since prolonged inhibition of protein synthesis can affect cell proteostasis [38]. For ERAD substrates retrotranslocation and degradation are often monitored by cell fractionation, deglycosylation or ubiquitination, and more recently exploiting split GFP- or Venus-based dislocation assay [39], [40]. Here we propose the use of Halotag to follow ER proteins degradation.

We first analysed whether the presence of a Halotag with or without a covalently bound ligand influenced µ_s_ degradation in HeLa transfectants (Figure 4B). To this aim, cells transfected with µ_s_ or Halo-µ_s_ were pulsed for 10 min with ^35^S. Where indicated, cells were treated with TMR before (16 hours) and during the radioactive pulse. Cells were then chased for different time points in a medium without TMR or radioactive amino acids and lysed in detergent. The anti-µ immunoprecipitates were resolved by SDS-PAGE under reducing conditions and transferred to nitrocellulose. Gels were first developed for autoradiography and then for TMR signal. As shown in Figure 4B, the degradation patterns of µ_s_ and Halo-µ_s_ are very similar, almost one third of radioactive µ_s_ present at time 0 being degraded after 4 hours of chase. Importantly, the presence of the ligand itself did not interfere with Halo-µ_s_ degradation. This is an important observation, since in all likelihood ERAD substrates are at least partially unfolded before being retro-translocated across the ER membrane and entirely before entry into the proteasomal cavity. The presence of a folded domain with covalently linked ligand could have impacted either one or both steps in the disposal of an ER luminal substrate. No other fragment containing the ligand can be seen in the blot (not shown), making very unlikely the possibility that endoproteases clipped the Halotag with the bound ligand off [41]. The degradation of the protein can also be seen directly by following the disappearance of the TMR fluorescence on the gel (red line in the graph). The decay observed in fluorescence is more rapid than the disappearance of the radioactive signal because in the 16 hours of staining with the Halo ligand also older molecules (closer to the dislocation-degradation steps) have been stained.

Taken together, these data show that the degradation of ERAD substrates can also be followed directly monitoring the disappearance of the TMR fluorescence.

### The Halo tag can be used to follow protein secretion

**Figure 5:**
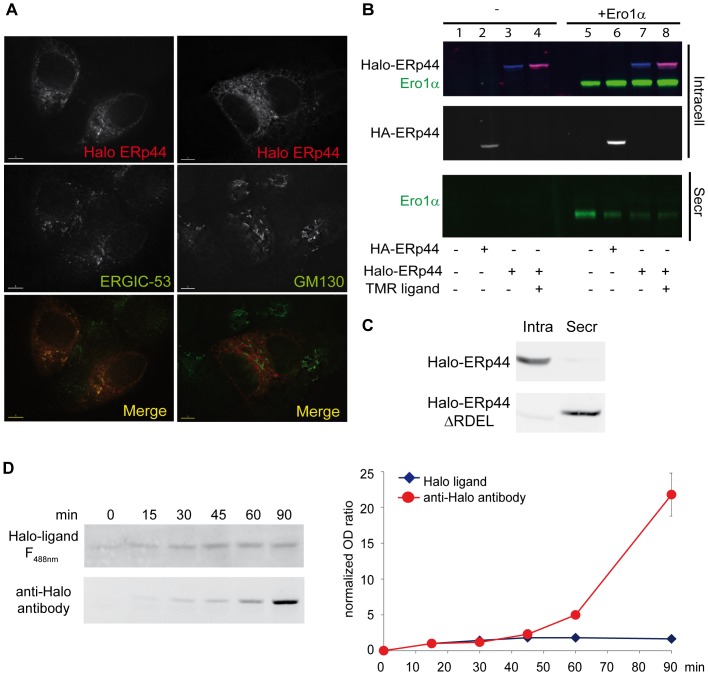
Halotag does not influence the function or localization of ERp44. **A** *Halo-tagging does not alter the localization of ERp44*. HepG2 cells were transiently transfected with Halo-ERp44 and pre-treated with TMR ligand (10 nM O/N) before fixation and staining with antibodies specific for ERGIC-53 or GM130 as markers of ERGIC or Golgi). Images were acquired with a 60X objective on an Olympus inverted fluorescence microscope and subjected to deconvolution. Single channel images and the merge are shown. Bar: 5 µm. **B** *Halo-ERp44 retains Ero1α efficiently*. Hela cells were transiently transfected with Halo-ERp44 and HA-ERp44 (alone or in combinations with Ero1α as indicated) 48 hours after transfection cells were washed and incubated for 4 hours in OPTIMEM. One sample was treated with TMR before and during the secretion. The spent media and cells were collected. The lysates corresponding to 10^5^ cells (lanes 1–4) and the TCA precipitated supernatants of 5 times as many cells (5×10^5^, lanes 5–8) were resolved under reducing conditions. Membranes were decorated with anti-Halo (blue signal), anti-HA (white signal), anti-Ero1α (green signal) and TMR fluorescence (red). Halo-ERp44 can be expressed and visualized by TMR staining. No significant differences were detected between Halo-ERp44 and HA-ERp44 in terms of function (Ero1α retention). **C** *Secretion of ERp44ΔRDEL is not impaired by appending a Halotag*. HeLa transfectants expressing Halo-ERp44 or Halo-ERp44ΔRDEL were incubated with OPTIMEM. After 4 hours, aliquots of lysates and supernatants (corresponding to 10^5^ cells) were resolved under reducing conditions and the blots decorated with anti-ERp44. As HA-ERp44 ΔRDEL [6], Halo-ERp44 ΔRDEL is massively secreted implying that the tag does not interfere with folding/secretion. **D** Hela cells transiently expressing Halo-ERp44ΔRDEL were incubated overnight with the R110 ligand (35 nM), washed and incubated in OPTIMEM (without the Halo-ligand) for the indicated time points. Supernatants corresponding to 5*10^6^ cells were concentrated by TCA precipitation, resolved under reducing conditions and the TMR signal collected using a fluorescent analyzer. The nitrocellulose filters were then decorated with anti-Halo antibodies. The intensities of the R110 fluorescent ligand and anti-Halo signals were quantified by densitometry using the ImageJ software and normalized to the value after 15 minutes of secretion. The graph shows the average of 2 independent experiments +/− standard deviation.

ERp44 cycles between the ER and cisGolgi and plays key roles in thiol-mediated protein quality control, redox homeostasis and calcium signalling [13], [42]. When expressed in mammalian cells, ERp44 prevents secretion of Ero1 flavoproteins and Peroxiredoxin 4, ER resident proteins which lack KDEL or other known localization motifs [12], [43], [44]. We inserted a Halotag between the cleavable ERp44 leader sequence and its first active sequence. Halo-ERp44 retained the subcellular distribution previously described for HA-tagged or untagged ERp44 [6], co-localizing mostly with the ERGIC marker ERGIC-53 and much less with GM130, a cisGolgi resident (Figure 5A). Halo-ERp44 can be easily visualized *in vivo*, staining cells with TMR (Figure 5A) or R110 (not shown). Moreover, Halo-ERp44 was as active as HA-ERp44 in retaining excess Ero1α (Figure 5B). Note how Western blots can be simultaneously stained with ligands and different antibodies emitting at different wavelengths, providing high quality, elegant images. The intracellular retention of ERp44 relies on its C-terminal RDEL motif [6]. We previously showed, using ^35^S radiolabeling [6], that deletion of the RDEL motif leads to rapid ERp44 secretion. As expected, also Halo-tagged ERp44ΔRDEL is massively secreted (Figure 5C), indicating that tagging does not interfere with folding or trafficking of the protein. The small molecular weight shift between intra- and extra-cellular Halo-ERp44ΔRDEL is due to O-glycosylation [45]. The velocity of secretion can be studied by using a protocol that resembles the ^35^S pulse and chase technique. HeLa cells transiently transfected with Halo-ERp44ΔRDEL were labelled with R110 ligand overnight. After several washes, the cells were incubated with a fresh medium without the ligand and the secreted material was then collected at different time points and analysed by SDS-PAGE. Consistently with our previous data, the pool of labelled Halo-ERp44ΔRDEL is completely secreted after 2 hours of chase, the curve reaching a plateau. On the contrary, the signal detected with anti-Halo antibodies (and hence representing labelled and non-labelled secreted ERp44ΔRDEL) kept steadily increasing, due to the addition to the release new molecules that are synthetized during the chase. Thus, the Halotag labelling is a valid alternative to the use of radioisotopes or cycloheximide treatment for studying protein secretion. Altogether our data indicate that the Halotag can be safely used to tag and follow an ER chaperone without disturbing its regulatory properties.

## Concluding Remarks

A significant advantage of the Halo domain is its ability to covalently bind ligands with tailored features. In this study, we exploited this property to visualise and quantify the traffic, condensation and degradation of different proteins within the early secretory pathway. First, we showed that the Halo moiety can correctly fold also in the oxidizing environment of the ER without forming aberrant intra- or inter-chain disulphide bonds. Moreover, no significant differences have been observed between ER-localized and cytosolic Halo for what concerns binding to its ligands (not shown). Our data also demonstrated that appending a Halo domain to different proteins had minor if any effects on the degradation, secretion or localisation of the resulting chimeras. Therefore, we could follow the fate of different proteins in the secretory pathway, without utilising radioactive isotopes or cycloheximide.

The most novel aspect of our work is the use of the Halotag technology to determine how potentially harmful protein deposits form and grow in the secretory pathway. We show here that aggregates formation over time proceeds as deposition of newly made proteins on a pre-existing condensation core, resulting in a progressive increase of the clusters size. In this external shell of the aggregates, no mixing between new and old material is observed, suggesting that µΔC_H_1 clusters are therefore rather “viscous” and grow mostly on the surface. However, given the limited resolution of confocal microscopy (200 nm laterally and 700 nm axially), we cannot determine if the newly deposited material can penetrate in the nucleation core of the RB.

By using live-cell imaging we also shed light on the mobility of Halo-µΔC_H_1 clusters. Our data indicate that, as expected, RB have on average a diffusion coefficient much lower than soluble ER proteins. Moreover, the mobility of big clusters is lower than that of small ones. A subset of RB shows a directional movement, likely due to microtubule transport. Accordingly, inhibiting microtubules decreased RB diffusion coefficient, indicating a partial role of microtubules in determining RB movements. Very interestingly, both small and big clusters displayed constrained mobility, which was not affected by Nocodazole. This observation suggests that an important constraining factor could be the ER membrane itself.

Numerous questions remain to be answered: do different seeds coalesce together? What determines their size? What is the structural organization of these clusters? If their content is viscous, how does it distribute concentrically? How do cells dispose of RB? Extending the techniques described herein to super-resolution fluorescence microscopy could allow answering many of these relevant issues.

Given the importance of protein aggregates, granules and clusters in storage diseases and also other regulatory processes [46], [47] our results describe a powerful tool for better visualizing and describing *in vivo* these events, being able to discriminate between old and young molecules in the same aggregate. Thus, this strategy is useful in order to reveal important mechanistic insights in the pathophysiology of ERSD and other disorders caused by proteotoxicity.

## Materials and Methods

### Reagents

Chemicals were from Sigma (St Louis, MO), unless otherwise indicated. Monoclonal anti-myc (9E10 clone) and anti-GM130 were used previously as described [48]. Polyclonal anti-PDI was a kind gift of Dr. Ineke Braakman (Utrecht, NL); goat anti-mouse IgG and IgM and anti-rabbit IgG (H+L) Alexa Fluor 700, 647, 546 and 488 were from Invitrogen Molecular Probes (Eugene, Oregon, USA); rabbit polyclonal anti-µ chain was from Zymed (San Francisco, CA). Cell permeant “no wash” TMRDirect and R110Direct ligands, and HaloTag Amine O4 ligand were from Promega (Promega Medison, WI).

### Plasmids construction

Vectors for the expression of HA-ERp44 and ERp44 no tag were previous described [48]. To obtain Halo-ERp44, ERp44 no tag was cleaved form pcDNA3.1(-) vector by XhoI and KpnI and inserted in pBlueScript II KS (+). A SgfI (GCG AT▾CGC) (Promega Madison, WI) site was inserted after the leader sequence cutting site by PCR (p44sgfI Fw and p44sgfI Rv primers). The PCR product was re-inserted in pcDNA 3.1 (-) vector by XhoI and KpnI. In pHTN HaloTag CMV-neo Vector, Halotag was mutagenized inserting a Sgf I site at the N terminal extremity by PCR (pHTN SgfI Fw and pHTN SgfI Rv primers) (Promega Medison, WI). Both the mutated constructs were digested by SgfI and purified by Wizard SV Gel and PCR Clean-Up System (Promega Medison, WI). Halo-ERp44 tagged was obtained by digestion by SgfI and ligation at 16°C over night from the sequence previously purified from agarose gel. The direction of the Halotag insertion was ascertained by SmaI digestion and the construct was sequenced (Primm s.r.l., Milan, Italy).

Halo-ERp44 ΔRDEL was obtained with digestion and ligation from the sequences of Halo-ERp44 and HA-ERp44 ΔRDEL: the 1007 Kbp fragment obtained by digestion of the plasmid HA-ERp44 ΔRDEL pcDNA3.1(-) with HindIII was exchanged with the one obtained from Halo-ERp44 pcDNA3.1(-) and checked by sequencing.

HaloRDRDEL was obtained by two sequential PCR starting from HaloERp44 construct. The first PCR was done to amplify Halo tag fragment (primers FW: ACGACTCACTATAGGGAGAC; RV: TTTTTTGGGGTACCTTATGCGGCCGCACCGGTGTTATCGCTCTGAAAGTAC). The PCR product was inserted into pcDNA3.1- vector by XbaI, KpnI digestion. The obtained construct was then processed by PCR to insert RDRDEL ER retention motif (primers FW: AGCGATAACACCGGTAGGGATCGAGATGAGCTTTAAGGTACCAAGCTT; RV: AAGCTTGGTACCTTAAAGCTCATCTCGATCCCTACCGGTGTTATCGCT). The construct was fully sequenced by Primm s.r.l.

Vectors for the expression of µ_S_ and µΔC_H_1 were previously described [8]. In pHTN HaloTag CMV-neo Vector, Halotag was mutagenized inserting Nru site at N terminal extremity and NotI site at C-terminal extremity by PCR. A pRS316 vector [49] µΔC_H_1 with cherry positioned after the V_H_ and the pHTN HaloTag PCR product were digested by Nru and NotI, purified by Wizard SV Gel (Promega Medison, WI) and ligated to obtain pRS316 vectors carrying V_H_-Halo-C_H_2 etc construct. This plasmid and pcDNA3.1(+) vectors carrying µ_S_ and µΔC_H_1 were digested by EcoRI and NotI to obtain respectively the V_H_-Halo and the C_H_1-C_H_4 and the C_H_2-C_H_4 moieties, purified by Wizard SV Gel (Promega Medison, WI) and ligated to obtain pcDNA3.1(-) vectors carrying µ_S_-Halo and µΔC_H_1-Halo. Halo Tag insertion direction was screened by EcoRI and XhoI digestion and checked by sequencing.

### Oligonucleotides

The following oligonucleotides were obtained from Primm s.r.l. Milano, Italy):

p44SgfI Fw, CCTGTAACAACTGAAATAGCGATCGCTGAAATAACAAGT;

p44SgfI Rv, ACTTGTTATTTCAGCGATCGCTATTTCAGTTGTTACAGG;

pHTN SgfI Fw, GCCGCGATCGCTGAAGCAGAAATCGGTAACTGGCTTTCCATTC;

pHTN SgfI Rv, GGAAGCGATCGCGTTATCGCTCTG.

### Cell culture, transfection and immunofluorescence

All the cell lines were obtained from ATCC and cultured and transfected by PEI (Polyethyleneimene, Polysciences inc. Warrington, PA) as previously described [50]. HepG2 cells were transfected by Fugene HD transfection reagent from Promega Corporation (Promega Medison, WI) following the manufacturer instructions.

For immunofluorescence analyses, cells were plated on 15 mm glasses and transfected. 48 hours after transfection cells were fixed with 4% PFA and processed as previously described [6]. For visualization with Halo-ligands, cells were treated O/N with the membrane permeable ligands TMR or R110 (both used at 10 nM). Samples were analyzed with a 60X objective on an Olympus inverted fluorescence microscope (model IX70) equipped with a quadriband dicroic filter (Sedat Quad, Chroma) with DeltaVision RT Deconvolution System (Alembic, HSR, Milano). After deconvolution, images were processed with Adobe Photoshop CS4 (Adobe Systems Inc.).

### Western blot

For Western blot assays, HeLa cells were detached by trypsinization and washed once in ice-cold phosphate buffered saline (PBS) and once in PBS with 10 mM N-ethylmaleimide (NEM) to block disulfide interchange [6]. Cells were then lysed in 150 mM NaCl, 1% NP-40, 0.1% SDS, 50 mM Tris HCl pH 8.0 (RIPA) containing 10 mM NEM and protease inhibitors for 20 min on ice. Aliquots of the postnuclear supernatants were analyzed by SDS PAGE and western blotting with monoclonal anti-ERp44 [6], anti-HA or polyclonal anti Halo (Promega). Signals were detected by infrared technology by FujiFilm (FLA 9000) (FujiFilm Life Science, Tokyo, Japan).

Lysates of HeLa cells transiently transfected with Halo-µ_s_ or Halo-µΔH1 were produced as described [8].

### Radioactive pulse and chase

Cells were incubated for 30 min in DMEM without methionine and cysteine supplemented with 1% dialysed FCS, pulsed with ^35^S-labeled amino acids (200 mCi/9*10^6^ cells) (Easy Tag, Perkin Elmer), washed and chased in complete medium for the indicated times. After different times, cells were treated with 10 mM NEM and lysed in RIPA as described [48]. Immunoprecipitates were resolved on SDS–PAGE under reducing conditions, transferred to nitrocellulose and membranes visualized by autoradiography with FLA900 Starion (FujiFilm Life Science, Tokyo, Japan). Subsequently, the red signal form TMR was visualized with infrared technology. Densitometric quantifications of the signals were performed with ImageJ.

### Confocal microscopy analysis: fluorescent pulse chase

For fluorescent pulse and chase experiments, Hela cells were seeded on 25 mm glasses before transfection with Halo-µΔC_H_1. The minimum incubation time necessary to have a signal in IF in our system with the “no-wash” TMRDirect ligand (5 µM) was 1 hour. However, in order to be sure to have completely saturated all the pre-existing molecules in the cells, 24 hours after transfection cells were stained for 24 hours with TMR (5 µM) in complete medium, extensively washed with PBS and then incubated with R110 (5 µM) in complete pre-warmed medium. After different chase times, cells were fixed in PFA, glasses mounted in glycerol and analyzed with a 60X objective with a Leica TCS SP2 Laser Scanning Confocal equipped with a spectral detector (Alembic, Ospedale San Raffaele, Milano, Italy). The green channel was excited with a 488 nm laser and, in order to avoid overlapping of the red channel, only the green emission spectra from 495 to 535 was acquired. The red channel was instead excited with a 543 laser and the emission spectra acquired was from 570 to 640 nm of wavelength. A single optical section, centered on the focal plane corresponding to the maximum size and brightness of the RB clusters was acquired for the quantification of the growth of the clusters. The size of the clusters was evaluated from the confocal images with a custom-written routine in Matlab, which allows the user to select the isolated clusters and plot the fluorescence intensity profile along the diameter of each cluster for both the green and the red channel. The diameter of the clusters is then measured as the full width at half maximum of the intensity profile and the relative cluster size increase is calculated as (*d*_g_–*d*_r_)/*d*_r_ where *d*_g_ and *d*_g_ are the cluster diameters measured in the green and in the red channel respectively.

### Confocal microscopy analysis: Live cell tracking of individual clusters

For live-imaging experiments, HeLa cells were seeded and transfected as above. 24 hours after transfection cells were labeled with 5 µM TMR ligand for 24 hrs. For Nocodazole treatment cells were incubated 9 hours with the TMR ligand 5 µM and 15 hours with 5 µM TMR ligand and 0.3 µg/ml Nocodazole. Cells were subsequently visualized with a Leica TCS SP5 Laser Scanning Confocal Microscope in phenol-free DMEM at 37°C and 5% CO2. A 543 nm laser was used for the excitation and the fluorescent light emitted from the sample was acquired with a 63x NA 1.4 oil immersion objective in the spectral window ranging from 560 nm to 644 nm. Time-lapse imaging was performed on individual cells by acquiring an image every 0.5 s seconds for 300 frames. We verified that no excessive photobleaching would perturb single particle tracking, by repetitive imaging of fixed samples using the same conditions as above: individual TMR molecules undergo photobleaching after more than 1000 frames on average, longer than our total acquisition time (data not shown).

To obtain tracking data on individual clusters, the time-lapse experiments were analyzed with the TrackMate plugin in Fiji [51]. Briefly, approximately 15 randomly chosen clusters were selected for each cell and tracked using the semi-automatic tracking tool in TrackMate. Tracks were then analyzed with custom-written routines in Matlab to quantify the MSD plot of each cluster. The first twenty time-points of the MSD curves were then fit by *MSD* = 4*Dt ^α^*, to obtain estimates for the short-term diffusivity *D* and for the anomalous diffusion exponent α. The Matlab routines are available at: https://github.com/shiner80/TM_export-ClustTrack.

## Supporting Information

Figure S1 **The Halotag can fold properly in the ER.** **A** HeLa cells were transfected with a cytosolic or an ER localized Halotag. Cells were stained O/N with the TMR ligand 5 µM and images acquired with an inverted microscope. Bar: 5 µm. **B** Lysates of HeLa cells expressing the ER-localized Halotag were loaded under reducing (R) and non-reducing (NR) conditions on a 3–8% polyacrylamide gradient gel. The Halo tag was detected with a polyclonal anti-Halo antibody. Note that no mobility shift is visible between the reducing and the non-reducing samples, indicating that the ER-localized Halo tag does not contain intra-chain or inter-chain disulfide bonds. **C** HeLa cells were transfected with a cytosolic or an ER localized Halotag and stained O/N with the TMR ligand 5 µM before lysis in RIPA buffer. Increasing amounts of lysates were loaded on reducing SDS-PAGE. First the signal of the TMR ligand was acquired; the filters were then decorated with a rabbit anti-Halo antibody and the signal of the secondary anti-Rabbit IgG antibody (Alexa 700) was then acquired. Densitometric quantifications are shown in the graph. Note that the signal of the TMR is much more linear and quantitative than the signal of the anti-Halo antibody.
(TIF)
