# Supplementary figures and images for: A Dynamic Study of Protein Secretion and Aggregation in the Secretory Pathway
Source: PLoS One. 2014 Oct 3;9(10):e108496. doi: 10.1371/journal.pone.0108496 (PMC4184786; doi:10.1371/journal.pone.0108496)

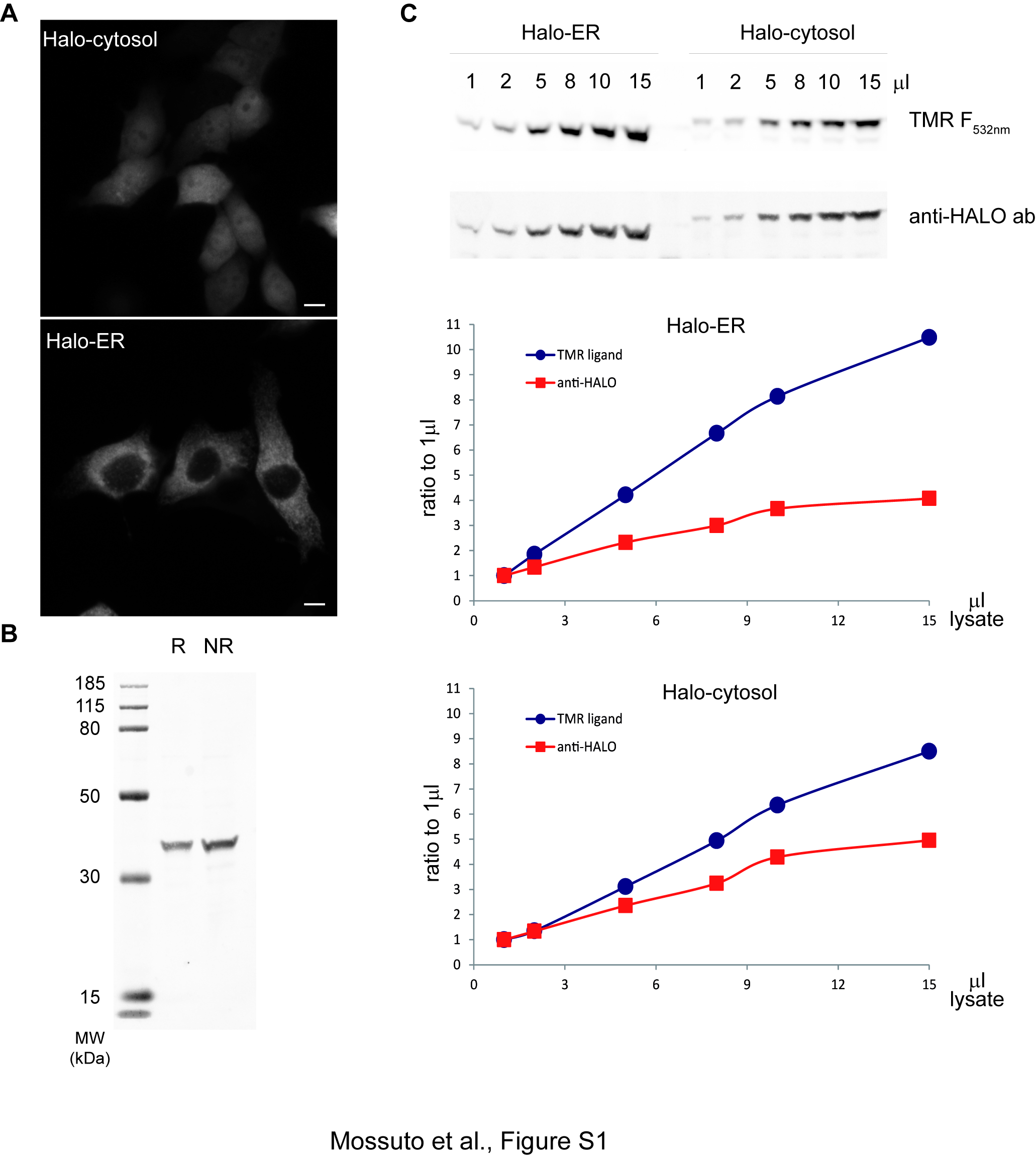

Supplement: Figure S1 — The Halotag can fold properly in the ER. A HeLa cells were transfected with a cytosolic or an ER localized Halotag. Cells were stained O/N with the TMR ligand 5 µM and images acquired with an inverted microscope. Bar: 5 µm. B Lysates of HeLa cells expressing the ER-localized Halotag were loaded under reducing (R) and non-reducing (NR) conditions on a 3–8% polyacrylamide gradient gel. The Halo tag was detected with a polyclonal anti-Halo antibody. Note that no mobility shift is visible between the reducing and the non-reducing samples, indicating that the ER-localized Halo tag does not contain intra-chain or inter-chain disulfide bonds. C HeLa cells were transfected with a cytosolic or an ER localized Halotag and stained O/N with the TMR ligand 5 µM before lysis in RIPA buffer. Increasing amounts of lysates were loaded on reducing SDS-PAGE. First the signal of the TMR ligand was acquired; the filters were then decorated with a rabbit anti-Halo antibody and the signal of the secondary anti-Rabbit IgG antibody (Alexa 700) was then acquired. Densitometric quantifications are shown in the graph. Note that the signal of the TMR is much more linear and quantitative than the signal of the anti-Halo antibody. (TIF) [file pone.0108496.s001.tif]
